# Evaluating traditional healers knowledge and practices related to HIV testing and treatment in South Africa

**DOI:** 10.1186/1472-698X-13-45

**Published:** 2013-10-23

**Authors:** Gavin George, Ethel Chitindingu, Jeff Gow

**Affiliations:** 1 Health Economics and HIV and AIDS Research Division (HEARD), University of KwaZulu-Natal, Durban, South Africa; 2 School of Commerce, University of Southern Queensland, Toowoomba, Australia

**Keywords:** Traditional healers, HIV and AIDS, HIV knowledge, South Africa

## Abstract

**Background:**

In a context of inadequate human resources for health, this study investigated whether traditional healers have the knowledge and skill base which could be utilised to assist in the scaling up of HIV prevention and treatment services in South Africa.

**Methods:**

Using a cross-sectional research design a total of 186 traditional healers from the Northern Cape province were interviewed. Responses on the following topics were obtained: socio-demographic characteristics; HIV training, experience and practices; and knowledge of HIV transmission, prevention and symptoms. Descriptive statistics and chi square tests were used to analyse the responses.

**Results:**

Traditional healers’ knowledge of HIV and AIDS was not as high as expected. Less than 50% of both trained and untrained traditional healers would treat a person they suspected of being HIV positive. However, a total of 167 (89%) respondents agreed using a condom can prevent HIV and a majority of respondents also agreed that having one sexual partner (127, 68.8%) and abstaining from sex can prevent HIV (145, 78.8%). Knowledge of treatment practices was better with statistically significant results being obtained.

**Conclusion:**

The results indicate that traditional healers could be used for prevention as well as referring HIV positive individuals for treatment. Traditional healers were enthusiastic about the possibility of collaborating with bio-medical practitioners in the prevention and care of HIV and AIDS patients. This is significant considering they already service the health needs of a large percentage of the South African population. However, further development of training programmes and materials for them on HIV and AIDS related issues would seem necessary.

## Background

South Africa has recently had a significant increase of HIV positive patients enrolled on anti-retroviral treatment (ART). In 2009, almost 90,000 children and 840,000 adults were enrolled in the public sector ART programme [1]. By 2011, ART was extended to 650,000 more people, increasing the total number on treatment to almost 1.6 million. The National Strategic Plan on HIV, STIs and TB (2012–2016) aims to have at least 80% of a total of 5.3 million HIV infected individuals on ART [1]. The burden of disease associated with untreated HIV and AIDS is significant and is particularly felt at rural bio-medical health facilities due to Human Resources for Health (HRH) shortages [2].

South Africa’s ratio of doctors per population is 50:100,000. The minimum requirement for a middle income country like South Africa is 180:100,000 according to World Health Organization (WHO) guidelines [3]. In South Africa it is estimated that of all doctor graduates, only about 3% end up working in rural areas [3]. With 40% of the population living in rural areas, HRH in rural bio-medical facilities are overburdened due to understaffing and the burden falls mainly onto nurses. This has negative implications; specifically, on the quality of HIV and AIDS services. Such challenges have prompted this research which aims to determine whether traditional healers (THs) are in a position to assist in HIV prevention and treatment services.

The ratio of THs is double (five per 1000) than that of bio-medical health care workers (2.3 per 1000) to the population in Africa [3]. In a context of an increasing burden of disease within South Africa and the subsequent HIV-related burden on public health facilities [4-6], THs, could potentially be utilised to alleviate this strain. Research indicates that mortality rates remain high amongst HIV positive individuals due to late initiation on treatment programmes [7]. Patients simply are not presenting themselves early enough to be initiated on treatment, especially in rural areas [7]. THs, who often are the initial health system contact for HIV patients, could refer them to the public sector treatment programme sooner, which could subsequently reduce morbidity rates.

Historically, the reliance on THs for health care has long been a norm amongst Black African people in sub-Saharan Africa. Data suggests that 80% of the Black African population consult THs for most of their health care needs [7-9]. The motivation behind the high rates of usage results from the focus on holistic healing offered by THs [10]. Holistic healing incorporates the healing of the mind, body, soul and restoration of harmony between the elements comprising the hierarchy of beings, the living and the living dead [8,11]. THs understand that an individual is relational, implying that “for good health, [the] individual and community must always be conscious of the symbiotic relationships between the living and the ancestors” [9].

Most Black Africans consult THs and bio-medical practitioners concurrently [9,10]. THs costs are usually much lower than bio-medical treatment [12]. Regardless, “most Africans know that African priests, …… political leaders, kings, chiefs and even presidents all occasionally consult a traditional doctor at critical times in their lives” [9]. THs have always been held in high esteem in African communities, due to their proximity to the community especially those in rural areas [13]. In contrast, the bio-medical health system is often difficult to access, due to the distance to the nearest facility and the associated costs of getting there [12,13]. Therefore, in the context of a lack of easily accessible bio-medical health facilities, high costs of transportation to these facilities and high rates of bio-medical staff attrition particularly in rural areas, THs could be used to assist in the HIV prevention and treatment effort.

It has been documented that over 60% of patients that THs attend to, have STIs and HIV and AIDS [13,14]. However, THs are seen by many in the bio-medical system as unfit to assist in the correct diagnosis of symptoms and prevention and treatment of HIV and AIDS [10]. Nurses were not willing to formally refer patients to THs whilst it was found that THs were open to referrals to public health facilities [10]. This resistance by bio-medical practitioners to collaborate has been attributed to differences in paradigms [10], the unscientific nature of methods used by THs, their unsystematic management of the healing process, and an unwillingness of THs to share their knowledge [15]. The stasis of bio-medical practitioners unwillingness to incorporate THs is concerning considering the increased burden of disease faced by health systems in Africa in the presence of the HIV epidemic.

In light of this situation, this paper attempts to answer the question as to whether THs are a suitable human resource to assist in the scaling up of HIV prevention and treatment services in South Africa. This involved assessing a sample of THs on their knowledge around effective HIV and AIDS prevention, diagnosis and treatment of symptoms and their willingness to interact with the bio-medical system.

## Methods

### Research design & sample

The study was implemented using a cross-sectional design. A total of 186 THs, comprising of 74 males and 112 females were interviewed.

### Survey frame, respondents and study site

The survey respondents were rural based THs who had met for a yearly meeting (indaba) organised by the Traditional Healing Organisation (THO) and had travelled to the meeting site of Kuruman from across the Northern Cape province of South Africa. All of the participants at the indaba were registered with the THO and willingly participated in the study.

### Survey instrument, implementation and analysis

A structured survey instrument was administered to this group of THs in February 2010. The instrument explored THs knowledge of HIV and AIDS, how they diagnosed it and their prevention and treatment practice.

The survey instrument consisted of four sections. The first section explored their socio-demographics characteristics; the second section explored their experience and training in traditional health practices. The third section explored their practice activity. The last section consisted of several psychometric scales to measure their knowledge on HIV transmission, prevention and symptoms. The technique for the psychometric knowledge scale was adapted from DeHart and Birkimer [16]. Some questions were worded in a positive direction (i.e. high score indicates high knowledge), and some negative (i.e. high scores indicates low knowledge). This was to prevent response bias. All scales were re-coded to ensure that a high score indicated high knowledge. The questions used in the knowledge scales were adapted from studies by the Human Sciences Research Council (HSRC) to allow for comparability with other sections of the South African population [17].

A pilot survey was conducted in Durban with five THs. This was primarily to pre- test the survey questions. However, it was also to discern the willingness of THs to share their practice information and opinions with researchers. Excellent cooperation was received in the pilot and this was replicated in the final survey data collection effort.

The final survey was translated from English into Setswana, the predominant language spoken in the Northern Cape. This was modified and back translated into English before being retranslated into Setswana. Following informed consent, respondents had the option of completing the survey themselves or it being administered to them by research assistants in either language. The majority of respondents chose the administered method with a large majority receiving it in Setswana.

### Data analysis

SPSS Version 21 was used for the data analysis. Descriptive statistics and chi-square tests were used to analyse the responses. Descriptive analysis was used to examine the socio-demographic characteristics of the sample. Using the chi-square test statistic, the association between having received HIV and AIDS training or not, healer’s knowledge of HIV and AIDS, HIV symptom identification as well as prevention behaviours and treatment practices were determined.

### Ethics

Ethical approval for this study was obtained from the University of KwaZulu-Natal’s Bio-medical Research Ethics Committee (Ethical Approval number: BF035/07).

## Results

### Demographics

In terms of racial composition, 185 were Black African and one was Mixed Race (Coloured). All except one participant were South African citizens. The majority (171) were Christians, whilst five stated that they belonged to the African animist tradition, and 10 participants did not record their religion. A large proportion (111, 60%) had previously undertaken HIV and AIDS training, with 75% (84) of those completing a five day THO developed and delivered HIV and AIDS training programme. 74 (40%) had not had any formal training in managing HIV and AIDS. In terms of educational qualifications, 171 had not progressed further than finishing primary school, 10 had attended high school and five had a diploma from a post-secondary programme.

### Traditional healers’ knowledge of HIV and AIDS

Respondents reported seeing on average seven patients per week whom they believed to have HIV. Many reported having good success in treating the symptoms of people living with HIV and AIDS with a majority claiming they were well known within the community for their ability to effectively treat HIV and AIDS symptoms.

In terms of THs treating patients they suspect of having HIV and AIDS, the results for those who had received training were not encouraging with a minority (51, 46%) willing to treat, whilst a majority either would not (20, 18%) or were unsure (40, 36%), if they would do so. Of those without any HIV training the results were similar with a minority (33, 44%) willing to treat, whilst the majority either would not (12, 16%) or were unsure (30, 40%), if they would do so.

A minority of respondents suggested they had a cure for HIV (see Table 1). The results for those who had received training were more encouraging than those who had no training, although this was not significantly different using a chi-square test (not reported here). On this question there was little ambiguity in responses with unsure responses being very insignificant. Respondents had strong views either way, with the vast majority of those who suspect there is a cure for HIV being quite certain about their response.

**Table 1:** **Treating patients with HIV and AIDS** (**n** = **186**)

**Question**	**Received training**	**Agree n**	**Agree %**	**Disagree or unsure n**	**Disagree or unsure %**
Do you treat patients who you suspect have HIV and AIDS?	Yes (111)	51	45.9%	60	54.1%
	No (75)	33	44.0%	42	56.0%
Do you think there is a cure for HIV and AIDS?	Yes (111)	40	36.0%	71	64.0%
	No (75)	32	42.6%	43	57.4%

Respondents were then asked about the transmission of HIV and AIDS to test their detailed knowledge levels. Having unprotected sex with a HIV positive person, as one of the ways of acquiring HIV, was statistically significant regardless of having received HIV training or not (see Table 2). Of those who had received training, 95 (84%) agreed that HIV could be transmitted by having unprotected sex with a HIV positive person. Of those without HIV training, 69 (93%) agreed with the statement and only three (4%) disagreed.

**Table 2:** **Knowledge of HIV** and **AIDS** (**n** = **186**, **α** = **0**.**05**)

**Question**	**Factor**	**Chi-square**	**DF**	***p-value***
Unprotected sex	HIV Training	7.982	2	0.018

Chi-square significance levels *p* < *0*.*05*.

The results for other questions on HIV knowledge showed mixed significance. Cross-tabulation results indicate 64 (58%) of those who had received and 40 (55%) who had not received previous training disagreed with the statement that HIV can be transmitted when a HIV person coughs or sneezes. When respondents were asked if one can get HIV from a mosquito bite, 51 (47%) of those who had training, disagreed. However, 48 (44%) of those who had not had previous training, agreed that HIV can be transmitted by a mosquito bite whilst 13 (18%) were unsure. When respondents were asked if one can get HIV from contact with body fluids of a person who is HIV positive, 42 (46%) of those who had previous training disagreed, whilst 43 (38%) agreed and 17 (15%) were unsure. Those who had previous training, 27 (37%) agreed, 27 (37%) disagreed and 19 (26%) were unsure. A total of 97 (86%) of those who had training and 62 (85%) who had no training agreed that HIV can be transmitted through blood transfusion. A total of eight (7%) trained respondents disagreed whilst five (6.8%) untrained respondents disagreed.

A majority agreed that HIV and AIDS can be prevented by having sexually transmitted illnesses treated, using condoms or abstaining. Majority (61%) of respondents were pro advocating the use of condoms. See Table 3.

**Table 3:** **HIV prevention** (**n** = **186**)

**One can prevent getting infected with HIV by**	**Agree n**	**Agree %**	**Unsure n**	**Unsure %**	**Disagree n**	**Disagree %**
Using contraceptives (the Pill)	23	12.6%	22	12.0%	138	75.4%
Getting a sexually transmitted infection treated	82	45.3%	33	18.2%	66	36.5%
Having only one sexual partner	127	68.6%	16	8.6%	42	22.7%
Abstaining from sex	145	78.8%	9	4.9%	30	16.3%
Using a condom	167	89.8%	10	5.4%	9	4.8%
**We should not expect people to use condoms because it is not part of African beliefs and culture**	50	27.3%	20	10.9%	113	61.7%

### Symptoms of HIV

Respondents were then asked what symptoms or combination of physical ailments alert them to a patient potentially being infected with HIV. It was found that weight loss was significant. More than half of the respondents (66%) who had received training agreed that weight loss was one of the symptoms that can forewarn them if a patient might be infected with HIV, whilst 36 (34%) disagreed. Of those who had not received previous training, 56 (80%) agreed that weight loss was one element that could alert them to a patient being infected with HIV and AIDS, whilst 14 (20%) disagreed.

Table 4 illustrates the significant results. No other questions on symptoms were significant. Both weight loss and bewitchment were statistically significant. A majority agreed that weight loss was a symptom of HIV. Also, a majority disagreed that bewitchment was a symptom of HIV.

**Table 4:** **HIV** and **AIDS symptoms** (**n** = **186**, **α** = **0**.**05**)

**Question**	**Factor**	**Chi-square**	**DF**	***p-value***
Weight loss	HIV Training	4.213	1	0.040
Bewitchment	HIV Training	7.086	1	0.008

Chi-square significance levels *p* < *0*.*05*.

Table 5 indicates that a majority agreed that skin lesions, diarrhoea or vomiting, TB and body wounds can be symptoms or form some combination of physical ailments that alert to HIV infection. However, most of the respondents disagreed with the view that epilepsy, bewitchment and red spots on the back of the neck were symptoms of HIV infection.

**Table 5:** **Physical ailments suggestive of HIV infection** (**n** = **186**)

**Question**	**Received training**	**Yes n**	**Yes %**	**No n**	**No %**
*Symptoms or combination of physical ailments alerting HIV infection*					
Skin lesions	**Yes**	68	64.8%	37	35.2%
	**No**	45	64.3%	25	35.7%
Diarrhoea or vomiting	**Yes**	69	65.7%	36	34.3%
	**No**	44	62.9%	26	37.1%
TB	**Yes**	60	57.1%	45	42.9%
	**No**	46	65.7%	24	34.3%
Epilepsy	**Yes**	24	22.9%	81	77.1%
	**No**	17	24.3%	53	76.0%
Bewitchment	**Yes**	9	8.6%	96	91.4%
	**No**	16	22.9%	54	77.1%
Red spots on back of neck	**Yes**	22	21.0%	83	79.0%
	**No**	18	26.0%	52	74.3%
Body wounds	**Yes**	55	52.4%	50	47.6%
	**No**	36	51.4%	34	48.6%

### Traditional healers’ knowledge of HIV and AIDS treatment

The majority (89%) who had HIV and AIDS training and also 55 (61%) of those who had no previous training agreed that HIV can become resistant if ART medication doses are missed. Of those trained, five (10%) were unsure and seven (8%) disagreed, whilst 11 (15%) untrained respondents were unsure and seven (10%) disagreed.

Majority (90%) of respondents who had received training agreed that ART medication strengthens the defenses of a person who has AIDS with eight (7%) unsure, and three (3%) who disagreed. A total of 57(78%) who had not received any previous training agreed, with nine (12%) unsure and seven (10%) disagreeing.

Respondents were further asked whether a person with AIDS must stay on ART even if s/he feels better. The responses to this question were significant with the majority who were trained (95%) agreeing, only one (1%) unsure and five (4%) who disagreed. Most (86%) THs who had no previous training also agreed, leaving seven (10%) unsure and three (4%) who disagreed.

The question about if a person with AIDS takes ART and whether s/he is more in control of his/her illness yielded significant results. The majority (94%) of trained respondents concurred with the statement whilst three (3%) were unsure, and a further three (3%) dissenting. Table 6 illustrates the significant results.

**Table 6:** **HIV** and **AIDS treatment** (**n** = **186**, **α** = **0**.**05**)

**Question**	**Factor**	**Chi-square**	**DF**	***p-value***
Medication doses missed	HIV Training	7.423	2	0.024
ART medication	HIV Training	5.837	2	0.050
Stay on ART medication	HIV Training	7.945	2	0.019
Control of illness	HIV Training	9.283	2	0.010

Chi-square significance levels *p* < *0*.*05*.

### Willingness to refer and collaborate

Respondents were asked whether they would be willing to refer patients to clinics or hospitals if they suspected them of being infected with HIV, with 75 (70%) of those who had received previous training confirming their willingness whilst 33 (30%) did not. Of those who had not received any previous HIV and AIDS training, 57 (83%) said yes whilst 12 (17%) said they were not willing to refer their patients to clinics/hospitals for HIV treatment. These results were significant. See Table 7.

**Table 7:** **HIV** and **AIDS patient referrals** (**α** = **0**.**05**)

**Question**	**Factor**	**Chi-square**	**DF**	***p-value***
Willingness to assist utilizing clinic or hospital services	HIV Training	3.848	1	0.050

Chi-square significance levels *p* < *0*.*05*.

## Discussion

Langlois-Kassen *et al*. estimates that THs already provide more services to people affected and infected with HIV and AIDS than the biomedical system [18]. This study’s primary research question was to determine THs suitability in assisting in the scaling up of HIV prevention and treatment services in South Africa. The results presented here indicate that they are already in regular contact with HIV positive individuals with the majority suggesting they treated an average seven patients per week.

The results indicate that THs have relatively low levels of HIV and AIDS knowledge but those who had received previous HIV and AIDS training had better outcomes than those without training. However, concern by biomedical practitioners is understandable given that some THs claim there is a cure for HIV (72 of 184). This result provides some justification for the views of bio-medical practitioners that THs do not possess the understanding and knowledge to effectively assist in HIV and AIDS management. However, THs who had previous training were knowledgeable about HIV and AIDS which suggests that THs can be educated further about its management. If they receive proper training, THs should be able to implement HIV prevention and care that is culturally appropriate and bio-medically correct [9,19]. There remains a willingness by the majority of THs to collaborate with the bio-medical system.

THs have always been revered in their communities due to the holistic nature of their care which includes in-depth counselling of their clients [10]. THs, due to their relationship with patients, could be used as advocates for HIV testing. Furthermore, due to their accessibility to the rural population they can influence the testing of patients earlier to reduce mortality rates due to late initiation on ART [20]. The majority of people fear finding out their status [1,21]. Bio-medical doctors often have inadequate time to provide in-depth counselling that is inclusive of the whole family. The western notion of testing and disclosure of results for HIV and AIDS has always been individualistic in that results are confidential. However, if THs are involved by bio-medical practitioners in providing counselling to the whole family or community, then it could be possible to reduce stigma and increase uptake of ART. Mbiti [22] stresses that the communal nature of Black African communities can be used as a strong support system that is advantageous to individuals infected with HIV and AIDS and potentially increase HIV testing levels. Nguni [19] found that THs, even when illiterate, are vital to disseminating information about the prevention of HIV and AIDS. Respondents revealed good knowledge on HIV prevention subjects. THs are trusted within the community, making it possible for them to discuss in-depth sexual issues and sensitive topics [19].

The results found that while the majority of trained respondents had higher levels of knowledge than those who hadn’t, most participants seemed to comprehensively understand what causes HIV transmission. Questions have been raised as to the validity of THs knowledge around HIV transmission [15], but they are already being consulted by patients believed to have HIV [23,24]. Integrating THs into HIV prevention and referral services will make it possible for collaboration with bio-medical practitioners. Collaboration is bound to bring about the most effective and efficient way of managing HIV and AIDS [23]. The development of training programmes and materials for THs on HIV and AIDS related issues would seem an important next step.

It still remains uncertain if bio-medical practitioners will acquiesce to having THs practicing in tandem with them. However, it seems that bio-medical practitioners will need a change of attitude to foster if they wish effective collaboration with THs. Colvin *et al*. [25] found TB patients were willing to consult a TH as their primary TB treatment supervisor with THs also willing to assist. The collaboration of THs with the biomedical system has been noted in other studies: Langlois-Klassen *et al*. [18] and Peltzer & Mngqundaniso [10].

### Limitations of the study

There is a possibility of selection bias due to the unique circumstances of the study which allowed the researchers to interview THs who attended the indaba. The profile and attitudes of those who did not attend the indaba may be very different from the interviewed respondents. The sample was also small considering the numbers of THs in South Africa. Lastly, the views of HIV positive patients are unknown because they were not interviewed.

## Conclusions

The South African population has been severely impacted by the HIV and AIDS epidemic. One of the outcomes has been to place an extra burden on the bio-medical system which is already struggling with insufficient human resources for health, especially doctors and especially in rural areas.

The results presented here suggest that THs are a suitable but under-utilized human resource for health which could support the biomedical system and mitigate the impact of HIV and AIDS. THs, due to their ability to provide culturally appropriate health services within communities are well suited to provide HIV prevention and treatment referral services.

Given the widespread usage of THs there could be real advantages to the community by utilizing them in supporting the work of the bio-medical system. Collaboration has long been advocated by the WHO [14,23]. Government and community bodies understand the need for more human resources for health to assist in scaling up effective services in HIV prevention and treatment. Therefore, there is a need to move from rhetoric to actual implementation, since THs are already engaged in mitigating the impact of HIV and AIDS in the community especially in the Black African population, and especially in areas not well resourced by bio-medical services. The development of training programmes and materials for THs on HIV and AIDS related issues would seem an important next step followed by the engagement of traditional healers and their representative organisations in professional development on this issue.

## Competing interests

The authors declare that they have no competing interests.

## Authors’ contributions

GG and JG designed the study. GG interpreted the analysis and planned the manuscript. EC undertook data analysis and contributed to writing manuscript. All authors read and approved the final manuscript.

## Authors’ information

**Gavin George** (Senior Researcher, Health Economics and HIV and AIDS Research Division (HEARD), University of KwaZulu-Natal, Durban, South Africa; Email: georgeg@ukzn.ac.za). **Ethel Chitindingu** (Researcher, Health Economics and HIV and AIDS Research Division (HEARD), University of KwaZulu-Natal, Durban, South Africa; Email: chitindingu@ukzn.ac.za). **Jeff Gow** (Professor, School of Commerce, University of Southern Queensland, Toowoomba, Australia; and Research Associate, Health Economics and HIV and AIDS Research Division (HEARD), University of KwaZulu-Natal, Durban, South Africa; Email: gowj@usq.edu.au).

## Pre-publication history

The pre-publication history for this paper can be accessed here:

http://www.biomedcentral.com/1472-698X/13/45/prepub
